# Increased Residual Force Enhancement in Older Adults Is Associated with a Maintenance of Eccentric Strength

**DOI:** 10.1371/journal.pone.0048044

**Published:** 2012-10-23

**Authors:** Geoffrey A. Power, Charles L. Rice, Anthony A. Vandervoort

**Affiliations:** 1 Canadian Centre for Activity and Aging, School of Kinesiology, Faculty of Health Sciences, The University of Western Ontario, London, Ontario, Canada; 2 Department of Anatomy and Cell Biology, The University of Western Ontario, London, Ontario, Canada; 3 School of Physical Therapy, Faculty of Health Sciences, The University of Western Ontario, London, Ontario, Canada; University of Alberta, Canada

## Abstract

Despite an age-related loss of voluntary isometric and concentric strength, muscle strength is well maintained during lengthening muscle actions (i.e., eccentric strength) in old age. Additionally, in younger adults during lengthening of an activated skeletal muscle, the force level observed following the stretch is greater than the isometric force at the same muscle length. This feature is termed residual force enhancement (RFE) and is believed to be a combination of active and passive components of the contractile apparatus. The purpose of this study was to provide an initial assessment of RFE in older adults and utilize aging as a muscle model to explore RFE in a system in which isometric force production is compromised, but structural mechanisms of eccentric strength are well-maintained. Therefore, we hypothesised that older adults will experience greater RFE compared with young adults. Following a reference maximal voluntary isometric contraction (MVC) of the dorsiflexors in 10 young (26.1±2.7y) and 10 old (76.0±6.5y) men, an active stretch was performed at 15°/s over a 30° ankle joint excursion ending at the same muscle length as the reference MVCs (40° of plantar flexion). Any additional torque compared with the reference MVC therefore represented RFE. In older men RFE was ∼2.5 times greater compared to young. The passive component of force enhancement contributed ∼37% and ∼20% to total force enhancement, in old and young respectively. The positive association (*R* ^2^ = 0.57) between maintained eccentric strength in old age and RFE indicates age-related mechanisms responsible for the maintenance of eccentric strength likely contributed to the observed elevated RFE. Additionally, as indicated by the greater passive force enhancement, these mechanisms may be related to increased muscle series elastic stiffness in old age.

## Introduction

Impaired force generating capacity is a consequence of natural adult aging resulting from many factors [1] including: the loss of contractile muscle mass [2], [3], decreased neural activation [4], [5], changes in muscle architecture [2] and excitation-contraction uncoupling [6]. Despite the age-related loss of isometric strength, during active lengthening (i.e., eccentric; ECC) of muscle, strength is well-maintained [7], [8]. Muscular force is dependent highly upon contractile history, such that isometric force of activated skeletal muscles following active stretch is greater than isometric force produced at the same muscle length prior to the stretch [9], [10]. This muscle property is termed residual force enhancement (RFE). Currently, it is unknown whether RFE is present in older adults and if the maintenance of ECC strength in old age contributes to RFE.

Age-related reductions in muscle contractile capacity are disparate when considered in terms of contractile mode [3]. The initial observation of Vandervoort et al. [8] reported older women had well-maintained knee extensor ECC strength compared with young [8]. This finding was later confirmed in older men [11] and other muscles [12], [13]. The maintenance of ECC strength with aging could relate to neural and mechanically mediated mechanisms. Neural factors could include: reduced agonist and increased antagonist activation during concentric actions thus reducing concentric strength, however older adults, if well practiced and accustomed with the task can fully activate their muscles, and antagonist coactivation is often not altered with aging [14], [15], [16], [17]. Therefore, the mechanism is likely related to the non-contractile and structural properties of the muscle tissue. Thus, an increase in muscle series elastic stiffness [18], [19] in older adults may yield greater passive resistance during muscle lengthening [11]. The elevated passive stiffness of muscle in old age could increase the effective ‘storage’ of elastic energy to optimise force production. Support of an intrinsic muscle property leading to maintained eccentric strength in older adults is the elevated tension (i.e. force) following a quick active stretch in single muscle fibers of older adults [20]. Elevated force could be related to increased force produced by individual cross-bridges during lengthening muscle actions. As well, the increased instantaneous stiffness in single muscle fibers from older adults observed by Ochala et al. [20] provides evidence of altered elastic, or structural properties of the muscle, independent of cross-bridge cycling responsible for the maintenance of ECC strength.

Residual force enhancement is evident in various muscle [10], [21], [22], [23], [24], [25] and human [26], [27], [28], [29], [30] preparations with active and passive properties of muscle force generating and transmitting structures suggested to contribute to RFE (for review see [31], [32], [33]). Mechanisms include: an increased proportion of strongly bound cross-bridges [34], increased average force produced by each cross-bridge [26] and the engagement of passive force (PFE) transmitting elements following stretch [35], [36] effectively ‘stiffening’ the contracting agonist thereby providing increased resistance to stretch. As well, RFE appears to be related, in part to the level of active muscle stiffness [37]. Noteworthy, the mechanisms of RFE following stretch appear to be similar to those responsible for maintenance of ECC strength in old age. Therefore, it is reasonable to propose RFE in older adults would be elevated compared with younger adults.

The purpose of this study is to utilize the aged muscle model to further explore RFE in a system in which isometric strength is compromised, but ECC strength is well-maintained. This study aims to determine whether RFE exists for the dorsiflexor muscles in older adults and to what extent as compared with young adults. We hypothesised steady-state isometric torque after active muscle stretch will be greater than the reference isometric steady state torque, in both absolute and relative terms, when compared with their younger counterparts. Thus, older adults will have greater RFE than young adults. As well, due to the age-related increase in muscle series elastic stiffness, passive force enhancement will be greater in older than younger adults following stretch.

## Materials and Methods

### Participants

All young (n = 10, 26.1±2.7y, 178.5±4.4 cm, 82.9±8.7 kg) and old men (n = 10, 76.0±6.5y, 174.4±5.9 cm, 82.8±10.4 kg) were asked to refrain from unaccustomed and strenuous exercise prior to testing and not to consume caffeine within 2 h prior to testing. All participants were recreationally active with no known neurological or musculoskeletal conditions. The young adults were recruited from the university population and the older adults were recruited from a local senior's fitness group which includes walking, light stretching and calisthenics 3 times per week. This study was approved by The University of Western Ontario Health Science Research Ethics Board for Research Involving Human Subjects and conformed to the Declaration of Helsinki. Informed verbal and written consent was obtained from all participants prior to testing.

### Experimental arrangement

All testing was conducted on a HUMAC NORM dynamometer (CSMi Medical Solutions, Stoughton, MA). The left foot was fastened tightly to the ankle attachment footplate with inelastic straps, aligning the lateral malleolus with the rotational axis of the dynamometer. Extraneous movements were minimized using non-elastic shoulder, waist and thigh straps. Participants sat in a slightly reclined position with the hip, and knee angles set at ∼110°, and ∼140° (180°; straight), respectively. All voluntary and evoked isometric dorsiflexion contractions were performed at an ankle joint angle of 40° of plantar flexion (PF). The ankle angle of 40° of PF was chosen to maximize the stretch of the dorsiflexor muscles during active lengthening. Lengthening contractions began at 10° PF until 40° of PF, and thus moved through a 30° joint excursion.

### Electromyography (EMG)

Electromyography signals were collected using self-adhering Ag-AgCl surface electrodes (1.5×1 cm; Kendall, Mansfield, MA). Prior to electrode placement, the skin was cleaned with pre-soaked alcohol swabs. The electrode configuration involved the active electrode positioned over the proximal portion of the tibialis anterior at the innervation zone (7 cm distal to the tibial tuberosity and 2 cm lateral to the tibial anterior border) [38] and a reference surface electrode was placed over the distal tendinous portion of the tibialis anterior just proximal to the level of the malleoli. The surface active electrode for the soleus was positioned 2 cm distal to the lower border of the medial head of the gastrocnemius and a reference was placed over the calcaneal tendon. The ground electrode was positioned over the patella. These electrode configurations were chosen to allow for a more global recording area.

### Experimental procedures

Stimulated contractions of the dorsiflexors were evoked electrically with two round carbon rubber electrodes (Empi, St. Paul, Minnesota, USA), coated in conductive gel positioned to maximize the twitch torque response, and secured with tape. The anode was positioned anterior and the cathode posterior to the fibular head over the deep branch of the common fibular nerve. A computer-triggered stimulator (model DS7AH, Digitimer, Welwyn Garden City, Hertfordshire, UK) set at 400V provided the electrical stimulation using a pulse width of 100 µs. Peak twitch torque (P_t_) was determined by increasing the current until a plateau in dorsiflexor P_t_ and tibialis anterior compound muscle action potential (M-wave) peak to peak amplitude were reached, and then the current was further increased by at least 15% to ensure activation of all motor axons via supramaximal stimulation. This stimulation intensity was used for the evoked doublet (P_d_) (two pulses at a 10 ms interpulse interval) to assess voluntary activation. Finally, a 10 and 50 Hz stimulus was delivered to assess low frequency torque depression and peak tetanic torque. For tetanic stimulation the current was increased until there was a plateau in evoked 50 Hz torque.

**Figure 1:**
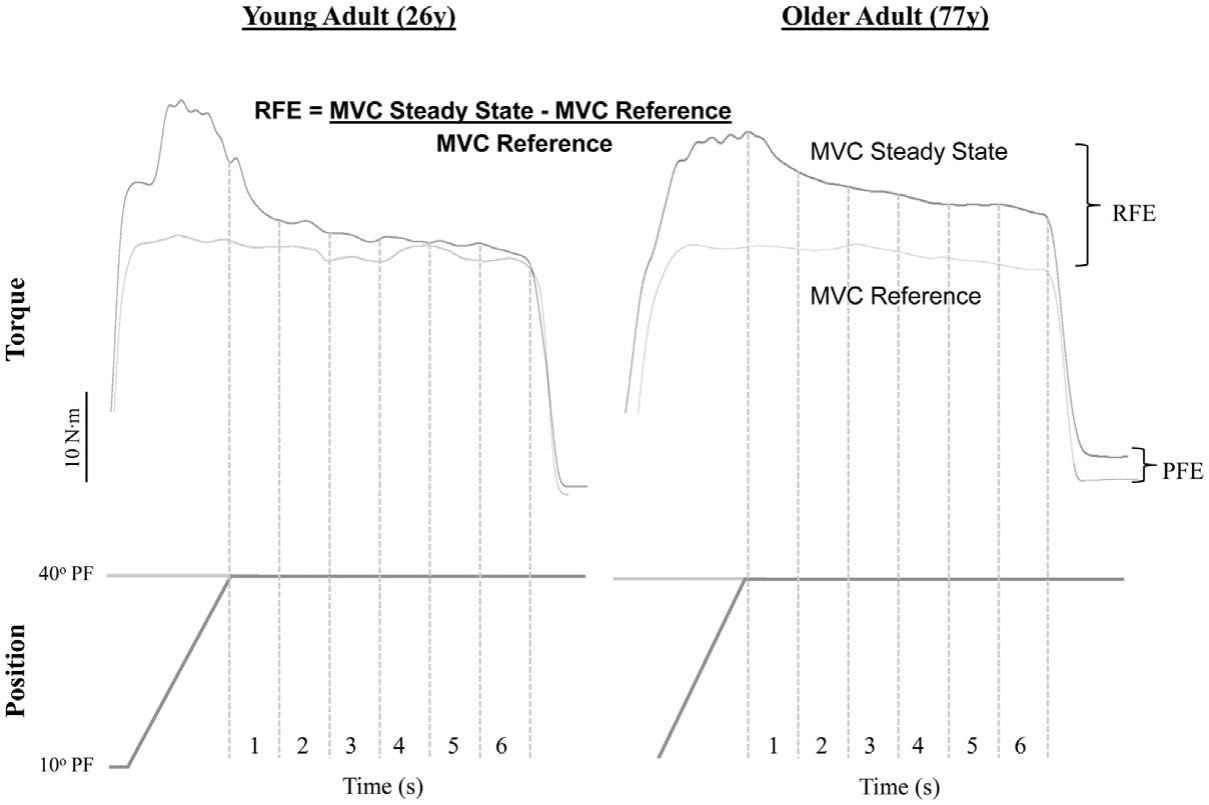
Raw data depicting the determination of residual force enhancement (RFE) in older and young men. Passive force enhancement (PFE) was determined as elevated force above rested baseline upon relaxation after stretch.

A 3–5s duration isometric dorsiflexor baseline MVC was performed to ensure there was sufficient time for subjects to reach peak MVC torque. During all MVCs, participants were provided visual feedback of the torque tracing on a computer monitor, and were exhorted verbally. Voluntary activation was assessed using the modified interpolated twitch technique [39]. The amplitude of the interpolated torque evoked during the peak plateau of the MVC was compared with a resting P_d_ evoked 1s following the MVC when the muscles were relaxed fully. Percent voluntary activation was calculated as voluntary activation (%)  = [1- interpolated P_d_/resting P_d_] ×100%. Values from the MVC with the highest peak torque were recorded. Contractile speeds were analyzed for twitch and 50 Hz tetanus, and time to peak twitch (TPT) was determined as the time for baseline torque to reach peak twitch torque. Half relaxation time of the twitch (HRT) and 50 Hz tetanus were determined as the time from peak torque amplitude to when half of the peak torque amplitude was reached. The protocol used to determine residual force enhancement (Figure 1) involved a 10 s isometric reference MVC at 40° of PF followed by 3 min of rest. The dorsiflexors were activated maximally for 10s, consisting of a 1s isometric contraction at the shortened muscle length (10° PF), followed by a 2 s lengthening stretch at 15°/s, and ending with a 7 s isometric MVC at the same ankle angle as the 10 s reference MVC (40° PF) (Figure 1). This sequence was repeated 2 times with the greater RFE value reported. Passive force enhancement (PFE) was calculated as the difference between resting torque after stretch and resting torque after a reference MVC (Figure 1). To compare the maximal strength of young and old during differing contraction types, eccentric strength (ECC) was determined as the maximum torque amplitude during stretch. After 3 min rest, a maximal voluntary shortening dorsiflexor contraction (15°/s) was performed to determine concentric (CON) strength.

### Data reduction and analysis

Torque and position data were sampled by the dynamometer at a rate of 500Hz. All data were converted to digital format using a 12-bit analog-to-digital converter (model 1401 Power, Cambridge Electronic Design, Cambridge, UK). Residual force enhancement was calculated by determining the mean torque value over 1s epochs during the last 6s of the 10s of the contractions. The values for the reference MVC, were divided into the mean torque value for each of the 6 1s epoch during the steady state MVC following the end of stretch, corresponding to the same time as the reference MVC (Figure 1). Steady state was defined as when the torque value following stretch reached a statistically significant difference from the first time epoch and force transients were no longer present. Residual force enhancement during steady state was defined as the percent increase in isometric torque following stretch, relative to the reference MVC.

Surface EMG signals were pre-amplified (×100), amplified (×2), band-pass filtered (10–1,000 Hz), and sampled online at 2500 Hz using Spike 2 software (version 7.07, Cambridge Electronic Design Ltd). The EMG signals for the tibialis anterior and soleus muscles were recorded during the dorsiflexor MVCs and expressed as a root mean square (RMS) value over each 1s epoch. Following stretch, EMG was analysed over each of the 6 1s epochs. Soleus EMG recorded during those periods was used to estimate antagonist coactivation as a soleus:tibialis anterior EMG ratio ×100%. All RMS values of EMG recorded from the tibialis anterior and soleus muscles for both the reference and steady state 10s contractions were normalized to the tibialis anterior and soleus EMG RMS values for the baseline agonist MVC. EMG from the trial which yielded the greater RFE was used for analysis. Post-activation potentiation was determined by calculating the ratio between the amplitude of the peak twitch torque recorded before and following the baseline isometric MVC. Spike 2 software (Version 7.07) was used off line to determine torque values of all contractions.

### Statistical analysis

**Figure 2:**
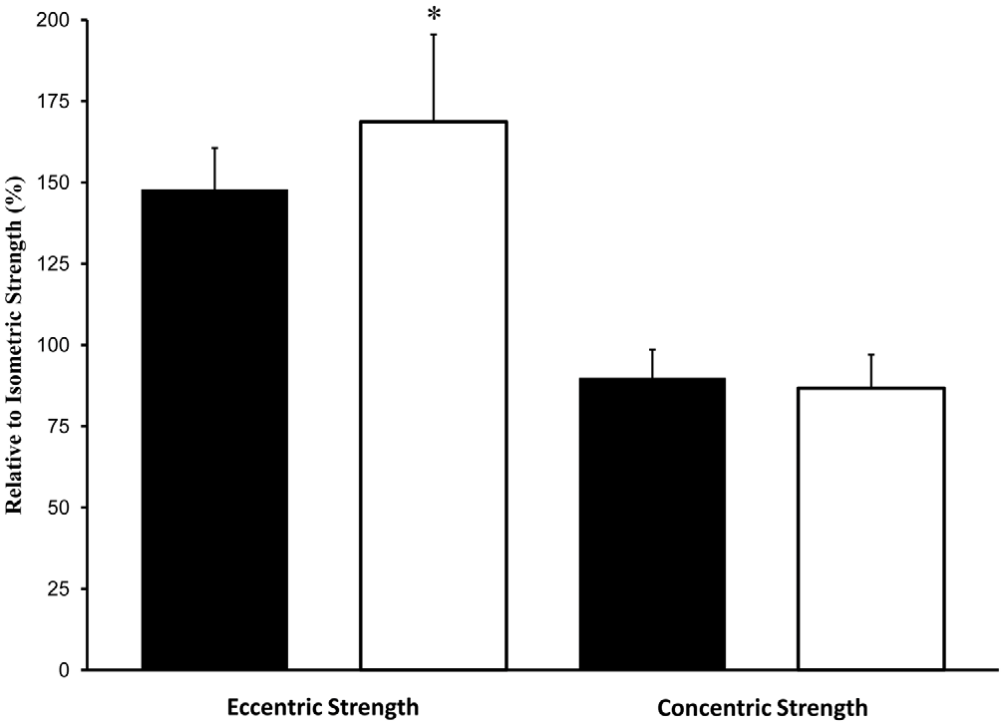
Eccentric and concentric strength in old (open bars) and young men (closed bars) relative to isometric strength. Values are Means ± standard error.

Using SPSS software (version 16, SPSS Inc. Chicago, IL) a one way analysis of variance (ANOVA) was performed to assess baseline neuromuscular function of the young and old adults. A two way ANOVA was performed to assess RFE between young and old over time. When significance was observed a *post hoc* analysis using unpaired t-tests was performed with a Bonferroni correction factor to determine where significant differences existed in RFE over time during the steady state. Voluntary activation values were not normally distributed, and thus a Mann-Whitney U-test was employed for this particular variable. The level of significance for all tests was set at *P*<0.05. A power calculation was determined to ensure there was sufficient power (1–β = 0.98–0.99) to detect significant differences. The tables are presented as means ± standard deviations (SD), and figure 2 as means ± standard error (SE). A Pearson correlation coefficient (*r*) and a linear regression analysis (*R* ^2^) were performed to evaluate the relationship and shared variance between the ratio of eccentric to concentric strength and residual force enhancement.

## Results

**Figure 3:**
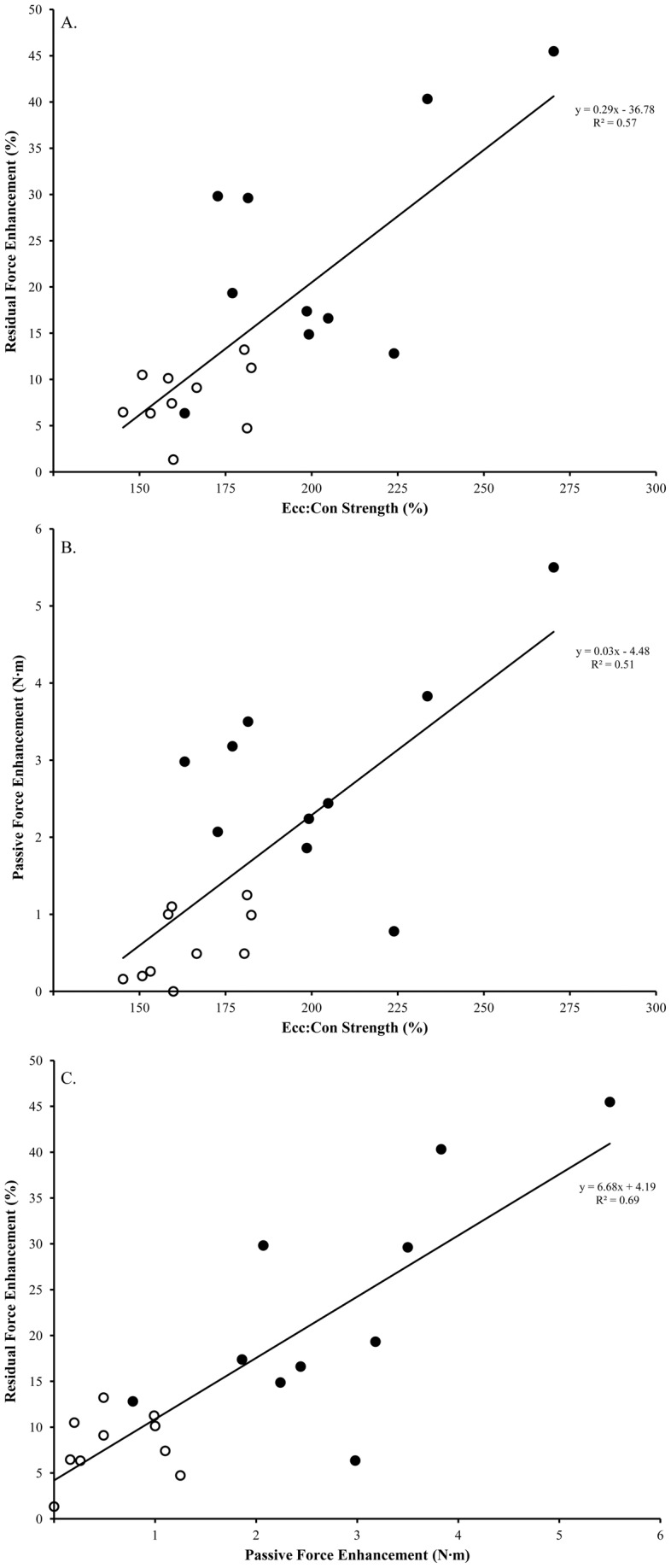
Relationship between the ratio of eccentric strength to concentric strength (Ecc:Con) and residual force enhancement (RFE) for old (closed circle) and young (open circle) (A.). Relationship between the ratio of eccentric strength to concentric strength (Ecc:Con) and passive force enhancement (PFE) for old (closed circle) and young (open circle) (B.). Relationship between the passive force enhancement (PFE) residual force enhancement (RFE) for old (closed circle) and young (open circle) (C.).

As shown in Table 1, older adults had a 26% lower twitch torque (*P*<0.05) compared to young, but both groups had similar evoked 10 Hz and 50 Hz torques (*P*>0.05). Contractile speeds for Pt and 50 Hz were 20–30% slower in the older adults compared with young. As well, the older adults had a 10% reduced capacity for potentiation compared to the young (*P*<0.05). During voluntary efforts, the old men were 13% weaker for isometric MVC torque (*P*<0.05) compared with the young men despite similar and equal high voluntary activations (∼98%, *P*>0.05) and similar levels of muscle co-activation (*P*>0.05) in both groups. Furthermore, concentric strength was 16% lower in older adults (*P*<0.05) compared to young. However, eccentric strength was well-maintained in the older men relative to isometric and concentric strength (*P*<0.05) and was not different to that reported for young (*P*>0.05) (Figure 2; Figure 3).

**Table 1:** Electrically evoked neuromuscular properties of the dorsiflexors.

Neuromuscular Properties of the Dorsiflexors									
		Electrically Evoked Isometric Properties							
Group	P_t_	TPT	HRT	Twitch	10Hz	50Hz	50Hz_HRT_	10∶50Hz	
(n = 10)	(N·m)	(ms)	(ms)	Potentiation	(N·m)	(N·m)	(ms)	(%)	
Young	5.3±1.2	110.7±15.7	109.5±22.7	119.8±15.6	14.8±3.1	21.5±5.1	138.2±26.7	0.69±0.11	
Old	3.9±1.2*	133.3±9.1*	146.0±23.9*	108.1±12.5*	14.5±3.2	20.0±5.3	173.2±19.0*	0.73±0.11	

Old men had lower absolute evoked peak twitch torque (P_t_), twitch potentiation (%), time to peak twitch (TPT), half-relaxation time (HRT) and 50Hz_HRT_ compared with young men. The 10Hz peak torque, 50Hz peak torque and 10∶50Hz ratio were not different between age groups. Values are means ± standard deviation. * Denotes significant age difference.

### Residual force enhancement

Peak eccentric torque values during active stretch were 70% and 50% greater than torque values reached during baseline isometric contractions in old and young (*P*<0.05), respectively (Table 2). Torque values were consistently higher in the steady state (isometric) phase following stretch for both old and young compared to the reference isometric MVC (Table 3; *P*<0.05). The steady state mean RFE for young and old was 8.4±5.0 and 21.7±13.5, respectively. Root mean square amplitudes of EMG for the tibialis anterior and co-activation of the soleus were similar for the isometric reference MVC and the steady state isometric phase following stretch in both old and young (*P*>0.05). Residual force enhancement was ∼2.5 times greater in the older adults compared to young (*P*<0.05) during the steady state isometric phase following stretch (Table 3). Both old and young adults experienced the typical exponential decay of torque to steady state following stretch (Figure 1); however the old had a longer time to steady state torque (2s) compared to young (1 s) (*P*<0.05). Passive force enhancement (PFE) (Table 2) was greater in older adults and contributed 37% and 20% to total RFE in old and young, respectively.

**Table 2:** Voluntary evoked neuromuscular properties of the dorsiflexors.

Neuromuscular Properties of the Dorsiflexors								
		Voluntary Contractile Properties						
Group	Voluntary	Isometric	Concentric	Eccentric			MVC	
(n = 10)	Activation	Strength	Strength	Strength	Ecc:Iso	Ecc:Con	Co- Act	PFE
	(%)	(N·m)	(N·m)	(N·m)	(%)	(%)	(%)	(N·m)
Young	99.6±1.3	33.2±6.4	29.9±4.7	48.5±7.3	147.8±12.9	162.9±13.1	22.6±6.5	0.59±0.45
Old	97.9±2.1	28.8±3.7*	25.0±4.6*	48.2±6.4	168.6±26.9*	199.2±32.8*	20.2±4.5	2.84±1.29*

Old men had lower maximal voluntary isometric contraction (MVC) strength and concentric strength. Voluntary activation and antagonist coactivation (MVC Co-act) was not significantly different between groups. Eccentric strength was well maintained in old men relative to young and other contraction modes (ratio of eccentric to isometric strength; Ecc:Iso, ratio of eccentric to concentric strength; Ecc:Con). Passive force enhancement (PFE) was greater in old than young. Values are means ± standard deviation. * Denotes significant age difference.

### Regression analysis

There was a positive association (*r* = 0.75, *P*<0.05) between the ratio of eccentric to concentric strength compared with RFE with a significant linear regression [RFE (%)  = 0.286× Ecc:Con strength – 36.78, *R^2^* = 0.57, *P*<0.05] (Figure 3A). Furthermore, there was a similar association (*r* = 0.71, *P*<0.05) between the ratio of eccentric to concentric strength compared with PFE with a significant linear regression [PFE (Nm)  = 0.034× Ecc:Con strength –4.48, *R^2^* = 0.51, *P*<0.05] (Figure 3B). Additionally, there was another positive association (*r* = 0.84, *P*<0.05) between PFE and RFE with a significant linear regression [RFE (%)  = 6.68× PFE (Nm) +4.19, R^2^ = 0.70, *P*<0.05] (Figure 3C). These relationships suggest that the muscle stiffness of older men leading to the maintenance of ECC strength in old age is paramount in explaining the elevated RFE in older compared to young adults.

## Discussion

We investigated the effects of natural adult aging on residual force enhancement (RFE) in a cross-sectional comparison of young and older men. Older men were weaker and slower for electrically evoked twitch properties, had blunted potentiation capacity and were weaker for isometric and concentric strength compared to young. However, despite impairments in these neuromuscular measures, eccentric strength was well-maintained in older adults and not different from the young. Residual force enhancement was calculated as the increase in torque during the isometric steady state following stretch compared to a reference MVC performed prior to stretch. In line with our hypothesis, older men had on average ∼2.5 times greater RFE during the isometric steady state following stretch compared to their younger counterparts, and passive force enhancement (PFE) contributed ∼37% and ∼20% to RFE for old and young, respectively. The impetus behind elevated RFE in older men appears to be related partly to the mechanisms responsible for the age-related maintenance of eccentric strength (Figure 3 A, B).

### Comparison with previous investigations of RFE in humans

Residual force enhancement was present in all young and older participants (Table 3). Force enhancement in the young (7–11%) was similar to values reported previously for voluntary contractions of the adductor pollicis 14% [26], plantar flexors 7–13% [27], and dorsiflexors 6–19% [27], [28], [30]. The present study is the first reported to investigate RFE in older adults and we found values ranging from 7–30%, with a mean of ∼25%. This level of RFE is considerably more than that reported for investigations on younger adults, with the exception of one study following exercise induced muscle damage in which RFE was elevated 150% above baseline values [30]. In older men, PFE contributed ∼17% more to RFE compared with young (Table 2), and thus PFE appears to be a key component of the overall increase in force production observed following active stretch, particularly in older adults (Figure 3C). After stretch, old and young followed the typical exponential decline in torque [32], but more time was required for the older men to reach a steady state torque level compared to young (Table 3). This longer time to steady state and elevated PFE indicates structural elastic mechanisms may have a disproportionately greater contribution to force enhancement in old age. Residual force enhancement in the present study is likely more related to mechanical factors compared to neural influences. Central activation (agonist; RMS EMG) levels were similar for the reference isometric MVC and steady-state MVC following stretch and differed minimally between young and older men. As well, antagonist coactivation was not significantly different between young and old, which is similar to previous findings for dorsiflexion contractions (14). Additionally, voluntary activation as assessed using the interpolated twitch technique [39] was near maximal and was similar for young and old. Thus, any confounding influence of age-related reductions in neuromuscular activation was avoided in the determination of RFE and the enhancement in older men likely can be attributed to age-related alterations within the muscle's structure.

**Table 3:** Residual force enhancement (RFE).

Time Following Stretch (s)	Absolute Residual Force Enhancement (N·m)		Relative Residual Force Enhancement (%)	
	Young	Old	Young	Old
**1**	5.0±1.4	9.5±3.7*	17.1±4.4	37.8±15.6*
**2**	3.2±1.1†	7.7±3.6*	11.2±4.6†	30.9±15.4*
**3**	2.8±1.2†	5.7±3.2*†	8.6±5.3†	22.7±13.1*†
**4**	2.3±0.9†	5.2±3.3*†	7.1±4.7†	20.9±14.3*†
**5**	1.7±0.9†	5.1±2.9*†	6.4±4.9†	20.7±12.8*†
**6**	1.9±1.2†	5.3±3.2*†	6.9±3.9†	22.4±14.0*†

Old men reached a steady state torque profile later than young men succeeding stretch and benefited from greater force enhancement in absolute and relative terms. Values are means ± standard deviation. * Denotes significant age difference. † Denotes steady state torque level.

For the determination of RFE, active stretch was initiated at 10° plantar flexion, which falls presumably on the ascending limb of the whole muscle force-length relationship for the dorsiflexors [40]. Stretch then continued to 40° of plantar flexion and at this muscle length most of the active sarcomeres would be operating near the plateau region of their length-tension relationship [40]. Residual force enhancement can be observed during stretch over the ascending limb of the force-length relationship [36], whereas PFE appears to be more dependent on activation over the descending limb in isolated preparations [36], [41] and humans [29]. Therefore, based upon the greater amount of PFE in old compared to young the force-length relationship may be different between young and older men. Passive force enhancement following muscle stretch after relaxation was present (Table 2) in every older participant (1.9–5.5 Nm) whereas the young participants displayed minimal passive force enhancement (0–1.3 Nm) upon relaxation. Because PFE is highly dependent upon stretch amplitude, the tibialis anterior of older men may lie further along the sarcomere length-tension curve. Thus, muscle of older men likely experienced greater stretch amplitude, which contributed to the greater RFE. However, this suggestion needs further investigation.

### Age-related maintenance of eccentric strength and elevated residual force enhancement

Mechanisms responsible for the age-related reduction in isometric strength, other than neural factors, can be attributed to intrinsic changes at the cellular and whole muscle level. Reduced force production of single muscle fibers with adult aging is likely related to the decrease in the number of viable cross-bridges and amount of force generated by each cross-bridge [42]. As well, excitation-contraction uncoupling contributes to impairments in force production [6] through failure to activate intact force generators. Following muscle stretch (i.e., isometric steady state) age-related decrements in cross-bridge function may be modified temporarily to improve isometric force generation which would contribute to elevated RFE compared to the young. With active stretch, there is potential for greater availability of actomyosin binding sites, allowing for the recruitment of weakly bound cross-bridges into a strongly bound cross-bridge state [20]. Therefore, the age-related decline in cross-bridge function could have been modified by stretch to increase the average force per cross bridge [43] contributing to RFE. Additionally, increased series elastic stiffness of the muscles of older adults, suggested to be an intrinsic muscle property leading to maintained eccentric strength in older adults [20], could also contribute to increased RFE in older men. Following a quick stretch the elevated tension (i.e. force) of fibers from older men may be related to increased force produced by individual cross-bridges during lengthening muscle actions [20], thereby increasing steady state force following stretch relative to reference MVC.

At the whole muscle level, age-related changes in muscle architecture contribute to force loss owing to shorter fascicle lengths and a less pennate fascicle organization, as well as increased tendon compliance (i.e. less stiff) [2], [44]. These changes in muscle architecture have functional implications on the overall musculotendinous unit. For example, during isometric contractions the greater tendon compliance in older men causes sarcomeres to shorten more compared to young, thus impairing their working length and force production by creating a state of less optimal myofilament overlap [45]. Hence, during isometric contractions the force generators work over a less optimal operational sarcomere length-tension range, impairing force production. Therefore, during the MVC steady state phase following stretch, the muscle from an older adult would potentially benefit from “taking up the slack” of the musculotendinous unit to optimize sarcomeric force production and effectively stiffening the series elastic complex for greater force transmission attenuating some of the age-related reduction in isometric force production. Furthermore, increased joint stiffness in old age [46], [47] and increased series elastic stiffness [18], [19] may have provided higher passive resistance during muscle lengthening [11] to optimise force production during the isometric steady state following stretch. Therefore, the engagement of passive series elastic structures in the muscle of older adults could contribute to greater overall force production as evident by the increased PFE in older compared to younger adults.

It seems in the present study that the passive component of force enhancement (i.e., PFE) contributed greatly to elevated force enhancement in older adults. Therefore, it appears that a system with compromised isometric force production such as natural aging, may share some similarities to lengthening-induced muscle damage model [30] in which the contribution of PFE to overall total force enhancement is markedly increased. The increased instantaneous stiffness following stretch in old muscle fibers observed by Ochala et al. [20] which contributes to the age-related maintenance of eccentric strength, contributes to elevated RFE in older adults. These findings support the role of an elastic structural mechanism of the muscle cell, independent of cross-bridge cycling contributing to RFE. Force enhancement following stretch has been attributed to the involvement of a passive structural element [25], [48] such as titin whose stiffness is increased by Ca^2+^ influx during force development in active muscle [24], [25]. In light of this, the greater PFE in older adults could be related to a potential age-related modification to titin, but this remains to be explored.

To explore possible mechanisms of residual force enhancement we utilized a model of natural human aging, in which age-related changes to the neuromuscular system yield impaired isometric strength while strength during lengthening actions is well maintained. Residual force enhancement is regarded as a fundamental property of muscle unaccounted for by the current swinging cross-bridge and sliding filament theory of muscle contraction. In the aged system, following stretch, RFE was elevated compared to young, possibly owing to increased cross-bridge function and the engagement of passive elements following stretch. Although the exact mechanisms cannot be elucidated at this time, stretch appeared to attenuate detrimental effects of aging on subsequent torque production and provided a mechanical strategy for enhanced muscle function over isometric actions.

## References

[1] Russ DW, Gregg-Cornell K, Conaway MJ, Clark BC (2012) Evolving concepts on the age-related changes in “muscle quality”. J Cachexia Sarcopenia Muscle 3: 95–109. 22476917 10.1007/s13539-011-0054-2 PMC3374023

[2] Narici MV, Maffulli N (2010) Sarcopenia: characteristics, mechanisms and functional significance. Br Med Bull 95: 139–159. 20200012 10.1093/bmb/ldq008

[3] Vandervoort AA (2002) Aging of the human neuromuscular system. Muscle Nerve 25: 17–25. 11754180 10.1002/mus.1215

[4] Aagaard P, Suetta C, Caserotti P, Magnusson SP, Kjaer M (2010) Role of the nervous system in sarcopenia and muscle atrophy with aging: strength training as a countermeasure. Scand J Med Sci Sports 20: 49–64. 20487503 10.1111/j.1600-0838.2009.01084.x

[5] Roos MR, Rice CL, Vandervoort AA (1997) Age-related changes in motor unit function. Muscle Nerve 20: 679–690. 9149074 10.1002/(sici)1097-4598(199706)20:6<679::aid-mus4>3.0.co;2-5

[6] Payne AM, Delbono O (2004) Neurogenesis of excitation-contraction uncoupling in aging skeletal muscle. Exerc Sport Sci Rev 32: 36–40. 14748548 10.1097/00003677-200401000-00008

[7] Roig M, Macintyre DL, Eng JJ, Narici MV, Maganaris CN, et al (2010) Preservation of eccentric strength in older adults: Evidence, mechanisms and implications for training and rehabilitation. Exp Gerontol 45: 400–409. 20303404 10.1016/j.exger.2010.03.008 PMC3326066

[8] Vandervoort AA, Kramer JF, Wharram ER (1990) Eccentric knee strength of elderly females. J Gerontol 45: B125–128. 2365961 10.1093/geronj/45.4.b125

[9] Abbott BC, Aubert XM (1952) The force exerted by active striated muscle during and after change of length. J Physiol 117: 77–86. 14946730 PMC1392571

[10] Rassier DE, Herzog W, Wakeling J, Syme DA (2003) Stretch-induced, steady-state force enhancement in single skeletal muscle fibers exceeds the isometric force at optimum fiber length. J Biomech 36: 1309–1316. 12893039 10.1016/s0021-9290(03)00155-6

[11] Hortobagyi T, Zheng D, Weidner M, Lambert NJ, Westbrook S, et al (1995) The influence of aging on muscle strength and muscle fiber characteristics with special reference to eccentric strength. J Gerontol A Biol Sci Med Sci 50: B399–406. 7583797 10.1093/gerona/50a.6.b399

[12] Porter MM, Vandervoort AA, Kramer JF (1997) Eccentric peak torque of the plantar and dorsiflexors is maintained in older women. J Gerontol A Biol Sci Med Sci 52: B125–131. 9060970 10.1093/gerona/52a.2.b125

[13] Poulin MJ, Vandervoort AA, Paterson DH, Kramer JF, Cunningham DA (1992) Eccentric and concentric torques of knee and elbow extension in young and older men. Can J Sport Sci 17: 3–7. 1322766

[14] Jakobi JM, Rice CL (2002) Voluntary muscle activation varies with age and muscle group. J Appl Physiol 93: 457–462. 12133850 10.1152/japplphysiol.00012.2002

[15] Klass M, Baudry S, Duchateau J (2005) Aging does not affect voluntary activation of the ankle dorsiflexors during isometric, concentric, and eccentric contractions. J Appl Physiol 99: 31–38. 15705734 10.1152/japplphysiol.01426.2004

[16] Roos MR, Rice CL, Connelly DM, Vandervoort AA (1999) Quadriceps muscle strength, contractile properties, and motor unit firing rates in young and old men. Muscle Nerve 22: 1094–1103. 10417793 10.1002/(sici)1097-4598(199908)22:8<1094::aid-mus14>3.0.co;2-g

[17] Power GA, Dalton BH, Rice CL, Vandervoort AA (2011) Power loss is greater following lengthening contractions in old versus young women. Age (Dordr). 2012 Jun; 34(3): 737-50. Available: http://www.ncbi.nlm.nih.gov/pubmed/21559865. 10.1007/s11357-011-9263-z PMC3337924 21559865

[18] Ochala J, Frontera WR, Dorer DJ, Van Hoecke J, Krivickas LS (2007) Single skeletal muscle fiber elastic and contractile characteristics in young and older men. J Gerontol A Biol Sci Med Sci 62: 375–381. 17452730 10.1093/gerona/62.4.375

[19] Hasson CJ, Miller RH, Caldwell GE (2011) Contractile and elastic ankle joint muscular properties in young and older adults. PLoS One 6: e15953. 21264315 10.1371/journal.pone.0015953 PMC3019216

[20] Ochala J, Dorer DJ, Frontera WR, Krivickas LS (2006) Single skeletal muscle fiber behavior after a quick stretch in young and older men: a possible explanation of the relative preservation of eccentric force in old age. Pflugers Arch 452: 464–470. 16622703 10.1007/s00424-006-0065-6

[21] Julian FJ, Morgan DL (1979) The effect on tension of non-uniform distribution of length changes applied to frog muscle fibres. J Physiol 293: 379–392. 315465 10.1113/jphysiol.1979.sp012895 PMC1280719

[22] Telley IA, Stehle R, Ranatunga KW, Pfitzer G, Stussi E, et al (2006) Dynamic behaviour of half-sarcomeres during and after stretch in activated rabbit psoas myofibrils: sarcomere asymmetry but no ‘sarcomere popping’. J Physiol 573: 173–185. 16527855 10.1113/jphysiol.2006.105809 PMC1618761

[23] Rassier DE, Pavlov I (2012) Force produced by isolated sarcomeres and half-sarcomeres after an imposed stretch. Am J Physiol Cell Physiol 302: C240–248. 21998143 10.1152/ajpcell.00208.2011

[24] Joumaa V, Leonard TR, Herzog W (2008) Residual force enhancement in myofibrils and sarcomeres. Proc Biol Sci 275: 1411–1419. 18348966 10.1098/rspb.2008.0142 PMC2602709

[25] Leonard TR, DuVall M, Herzog W (2010) Force enhancement following stretch in a single sarcomere. Am J Physiol Cell Physiol 299: C1398–1401. 20844251 10.1152/ajpcell.00222.2010

[26] Lee HD, Herzog W (2002) Force enhancement following muscle stretch of electrically stimulated and voluntarily activated human adductor pollicis. J Physiol 545: 321–330. 12433972 10.1113/jphysiol.2002.018010 PMC2290659

[27] Pinniger GJ, Cresswell AG (2007) Residual force enhancement after lengthening is present during submaximal plantar flexion and dorsiflexion actions in humans. Journal of applied physiology 102: 18–25. 16946022 10.1152/japplphysiol.00565.2006

[28] Tilp M, Steib S, Herzog W (2009) Force-time history effects in voluntary contractions of human tibialis anterior. Eur J Appl Physiol 106: 159–166. 19214557 10.1007/s00421-009-1006-9

[29] Shim J, Garner B (2012) Residual force enhancement during voluntary contractions of knee extensors and flexors at short and long muscle lengths. J Biomech 45: 913–918. 22356842 10.1016/j.jbiomech.2012.01.026

[30] Power GA, Rice CL, Vandervoort AA (2012) Residual force enhancement following eccentric induced muscle damage. J Biomech 45: 1835–1841. 22542219 10.1016/j.jbiomech.2012.04.006

[31] Campbell SG, Campbell KS (2011) Mechanisms Of Residual Force Enhancement In Skeletal Muscle: Insights From Experiments And Mathematical Models. Biophys Rev 3: 199–207. 22180761 10.1007/s12551-011-0059-2 PMC3237401

[32] Edman KA (2012) Residual force enhancement after stretch in striated muscle. A consequence of increased myofilament overlap? J Physiol 590: 1339–1345. 22331422 10.1113/jphysiol.2011.222729 PMC3382324

[33] Rassier DE (2012) The mechanisms of the residual force enhancement after stretch of skeletal muscle: non-uniformity in half-sarcomeres and stiffness of titin. Proc Biol Sci 279: 2705–2713. 22535786 10.1098/rspb.2012.0467 PMC3367795

[34] Rassier DE, Herzog W (2004) Considerations on the history dependence of muscle contraction. Journal of applied physiology 96: 419–427. 14715673 10.1152/japplphysiol.00653.2003

[35] Edman KA, Elzinga G, Noble MI (1982) Residual force enhancement after stretch of contracting frog single muscle fibers. J Gen Physiol 80: 769–784. 6983564 10.1085/jgp.80.5.769 PMC2228643

[36] Herzog W, Leonard TR (2002) Force enhancement following stretching of skeletal muscle: a new mechanism. J Exp Biol 205: 1275–1283. 11948204 10.1242/jeb.205.9.1275

[37] Rassier DE, Herzog W (2005) Relationship between force and stiffness in muscle fibers after stretch. J Appl Physiol 99: 1769–1775. 16002777 10.1152/japplphysiol.00010.2005

[38] Botter A, Oprandi G, Lanfranco F, Allasia S, Maffiuletti NA, et al (2011) Atlas of the muscle motor points for the lower limb: implications for electrical stimulation procedures and electrode positioning. Eur J Appl Physiol 111: 2461–2471. 21796408 10.1007/s00421-011-2093-y

[39] Gandevia SC (2001) Spinal and supraspinal factors in human muscle fatigue. Physiol Rev 81: 1725–1789. 11581501 10.1152/physrev.2001.81.4.1725

[40] Maganaris CN (2001) Force-length characteristics of in vivo human skeletal muscle. Acta Physiol Scand 172: 279–285. 11531649 10.1046/j.1365-201x.2001.00799.x

[41] Herzog W, Leonard TR (2005) The role of passive structures in force enhancement of skeletal muscles following active stretch. J Biomech 38: 409–415. 15652538 10.1016/j.jbiomech.2004.05.001

[42] D'Antona G, Pellegrino MA, Adami R, Rossi R, Carlizzi CN, et al (2003) The effect of ageing and immobilization on structure and function of human skeletal muscle fibres. J Physiol 552: 499–511. 14561832 10.1113/jphysiol.2003.046276 PMC2343394

[43] Mehta A, Herzog W (2008) Cross-bridge induced force enhancement? J Biomech 41: 1611–1615. 18387614 10.1016/j.jbiomech.2008.02.010

[44] Narici MV, Maganaris CN, Reeves ND, Capodaglio P (2003) Effect of aging on human muscle architecture. J Appl Physiol 95: 2229–2234. 12844499 10.1152/japplphysiol.00433.2003

[45] Narici MV, Maffulli N, Maganaris CN (2008) Ageing of human muscles and tendons. Disabil Rehabil 30: 1548–1554. 18608375 10.1080/09638280701831058

[46] Vandervoort AA, Chesworth BM, Cunningham DA, Paterson DH, Rechnitzer PA, et al (1992) Age and sex effects on mobility of the human ankle. J Gerontol 47: M17–21. 1730848 10.1093/geronj/47.1.m17

[47] Winegard KJ, Hicks AL, Sale DG, Vandervoort AA (1996) A 12-year follow-up study of ankle muscle function in older adults. J Gerontol A Biol Sci Med Sci 51: B202–207. 8630696 10.1093/gerona/51a.3.b202

[48] Herzog W, Duvall M, Leonard TR (2012) Molecular mechanisms of muscle force regulation: a role for titin? Exerc Sport Sci Rev 40: 50–57. 22295281 10.1097/JES.0b013e31823cd75b

